# Simultaneous quantification of anti-Spike IgG subclasses against various SARS-CoV-2 variants using a multiplex serological assay

**DOI:** 10.1007/s00430-026-00878-y

**Published:** 2026-05-22

**Authors:** Marie L. Bischof, Pascal Irrgang, Matthias Tenbusch, Oliver T. Keppler, Paul R. Wratil

**Affiliations:** 1 Max von Pettenkofer Institute & Gene Center, Virology, National Reference Center for Retroviruses, LMU München, Munich, Germany; 2 Institut für klinische und molekulare Virologie, Universitätsklinikum Erlangen und Friedrich-Alexander-Universität (FAU) Erlangen-Nürnberg, Erlangen, Germany; 3 Medical Immunology Campus Erlangen, Friedrich-Alexander-Universität (FAU) Erlangen-Nürnberg, Erlangen, Germany; 4 German Center for Infection Research (DZIF), Partner Site Munich, Munich, Germany; 5 Faculty of Medicine, Max von Pettenkofer Institute & Gene Center, Virology, LMU München, Feodor-Lynen-Straße 23, 81377 Munich, Germany

**Keywords:** Electrochemiluminescent immunoassay, IgG subclasses, SARS-CoV-2, COVID-19, Humoral immunity

## Abstract

Recent research indicates that the composition of serum anti-SARS-CoV-2 Spike IgG subclass antibodies changes upon repeated exposure to viral Spike. These antibody subclass dynamics were suggested to impact adaptive immune responses and protection against infection. To characterize the role of the IgG subclass composition in the immune response to SARS-CoV-2, assays that measure virus variant-specific anti-Spike IgG subclass concentrations are needed. Here, we describe an easy-to-use serological method for quantifying anti-Spike IgG subclass antibodies against various SARS-CoV-2 variants in multiplex from less than 10 µL of serum. Our assay enables antigen-independent quantification, making it easily adaptable to emerging variants or other pathogens. We validated our method with 20 sera collected from 10 infection-naïve individuals after the second and third COVID-19 mRNA vaccination. We observed increasing anti-Spike IgG2 and, especially, IgG4 concentrations after the third immunization. More recent Spike variants preserved this pattern with decreased levels of total anti-Spike IgG. Our results showed a strong positive correlation with those from an established flow cytometry-based approach. Our method might help deepen our understanding of immune responses to SARS-CoV-2 and, moreover, help gauge the individual’s protection from severe disease.

**Supplementary Information:**

The online version contains supplementary material available at 10.1007/s00430-026-00878-y.

## Introduction

mRNA vaccines are a revolutionary class of drugs that was applied for the first time on a large scale in response to the coronavirus-disease 2019 (COVID-19) pandemic. Both approved mRNA vaccines, i.e. BNT162b2 and mRNA-1273, encode the Spike protein of the severe acute respiratory syndrome coronavirus 2 (SARS-CoV-2) as their immunogen and were shown to evoke immune responses that significantly increase protection against severe disease [1, 2].

A correlate of vaccine-induced protection against symptomatic and severe COVID-19 are neutralizing antibodies [3]. Among these, immunoglobulin G (IgG) antibodies are especially important to exert neutralizing functions in the blood. Mainly in research settings, total levels of serum anti-Spike IgG concentrations are, therefore, frequently measured to estimate protection against COVID-19. There are, however, four IgG subclasses (IgG1 – IgG4) that differ in their capacity to mediate effector functions, including antigen-dependent complement deposition and phagocytosis. In case of immunity to viral infections, there is evidence that IgG antibodies of subclass 2 and 4 induce, overall, the weakest effector functions whereas IgG1 and IgG3 constitute the most potent subclasses [4, 5].

Interestingly, multiple studies have reported an increase in pathogen-specific IgG4 after the third consecutive vaccination with an mRNA vaccine which did not occur after repeated vaccination with an adenoviral vector-based vaccine [6–13]. While virus neutralization capacity was not impaired in three-times mRNA-vaccinated individuals, high proportions of anti-Spike IgG4 were found to be associated with a decrease in complement deposition and phagocytotic activity [6], as well as diminished antibody dependent cellular cytotoxicity [8, 14]. Furthermore, a higher ratio of anti-Spike IgG4 + IgG2 concentrations to IgG1 + IgG3 concentrations was described as a risk factor for SARS-CoV-2 infection [15]. Based on these findings, dissecting the IgG subclass composition of vaccine- and infection-induced anti-Spike IgG antibodies could help expand our knowledge of SARS-CoV-2-specific immunity. Due to the ongoing evolution of the SARS-CoV-2 Spike antigen [16], measuring virus variant-specific anti-Spike IgG subclass compositions may be even more insightful.

Until today, ELISA-based methods and more complex multiplex techniques were applied to measure the anti-Spike IgG composition [6, 7, 9, 10, 14, 17–19]. A common caveat of these techniques is that they are relatively low throughput and often only cover a single variant’s antigen. Additionally, the quantification of the antigen-specific IgG subclass concentrations in these assays is tedious because it relies on the use of recombinant monoclonal antibodies that have the same Spike binding epitope but are of different subclasses [6, 14]. Thus far, no method to measure anti-Spike IgG subclass responses against several different virus-variants was described that does not rely on the use of such recombinant monoclonal antibodies.

Here, we report the establishment of an easy-to-use assay that can be set up for high-throughput measurements to quantify serum compositions of IgG subclasses against the Spike receptor binding domain (RBD) of up to nine different SARS-CoV-2 variants in multiplex. The assay was designed in a 96-well plate format, and it follows the principle of an electrochemiluminescent immunoassay. In contrast to other techniques, our assay quantifies subclass-specific anti-Spike antibody concentrations independently of the coated antigen. This makes our method easy to implement and, furthermore, adaptable for future SARS-CoV-2 variants as well as other pathogens.

## Materials and methods

### Anti-Wuhan Hu-1 Spike S1 IgG antibody quantification

Anti-Wuhan Hu-1 Spike S1 antibody concentrations in sera were quantified using the commercial anti-SARS-CoV-2 IgG QuantiVac-ELISA (EuroImmun, Cat.: EI 2606-9601-10 G) according to the manufacturer’s instructions. Briefly, 100 µL of calibrators and 100 µL of 1:101 diluted serum samples were incubated for 1 h at 37 °C. Afterwards, the plates were washed and 100 µL of conjugate was added followed by another incubation step for 30 min at 37 °C and subsequent washing. Finally, 100 µL of substrate was added and the plates were incubated for 30 min in the dark at 25 °C. The reaction was stopped by addition of 100 µL of stopping solution. The absorbance of the reaction product was recorded in a plate reader within 30 min after stopping. In accordance with the manufacturer’s recommendations, samples that gave “out of range” signals were diluted further in sample buffer and measured again until quantifiable results were obtained. Following the manufacturer’s protocol, kit-internal calibrators (previously validated with the WHO IVIg standard, NIBSC: 20–136) were measured and the signals obtained from these calibrators were utilized to calculate the anti-Wuhan Hu-1 Spike S1 concentrations in binding antibody units (BAU)/mL, in the serum specimens.

### Anti-Spike IgG subclass characterization

The V-PLEX SARS-CoV-2 Key Variant RBD Panel 1 Kit (Meso Scale Diagnostics (MSD), Cat.: K15659U (IgG)) and the V-PLEX SARS-CoV-2 Panel 24 Kit (MSD, Cat.: K15575U (IgG)) were used to characterize the IgG subclass composition of anti-Spike antibodies. These commercially available kits contained wash buffer (MSD, Cat.: R61AA-1), sample diluent (MSD, Cat.: R50AA), blocking solution (MSD, Cat.: R93AA), and read buffer (MSD, Cat.: R60AM), as well as plates pre-coated with 10 spots per well each containing a different SARS-CoV-2 antigen. The antigen-coated plate was prepared by blocking with 150 µL/well of blocking solution for 30 min. Subsequently, the wells were washed three times with 150 µL/well wash buffer. Samples were first diluted in diluent to an anti-Spike IgG concentration of 5 BAU/mL previously determined by the commercial anti-SARS-CoV-2 IgG QuantiVac-ELISA. After performing serial dilution experiments with the participants’ sera, we observed that at this concentration the signals detected under all conditions were below saturation indicating an excess of unbound antigen (suppl. Figure 1). 50 µL of each diluted sample were added in quadruplicates to the plate and incubated for 2 h. After incubation with the sera, the plates were washed three times with 150 µL/well, and then 50 µL/well of the respective biotinylated secondary antibody was added. The following secondary antibodies and concentrations were utilized: 1.9 µg/mL mouse anti-human IgG1 clone 8c/6–39 (Sigma-Aldrich, Cat.: B6775), 0.1 µg/mL mouse anti-human IgG2 clone HP6002, (Southern Biotech, Cat.: 9070-08), 2 µg/mL rabbit anti-human IgG3 clone RM119 (Thermo Scientific, Cat.: MA5-27933), and 0.1 µg/mL mouse anti-human IgG4 clone HP6023, (Southern Biotech, Cat.: 9190-08). Following 1 h incubation with the secondary antibodies, the plates were washed again three times with 150 µL/well washing buffer. Next, 25 µL/well SULFO-tag streptavidin (MSD, Cat.: R32AD) at 0.2 µg/mL in diluent were added and the plates were incubated for 1 h. Prior to reading, the plates were washed again three times with 150 µL/well washing buffer. Finally, 150 µL/well read buffer was added and measurements were performed on a MESO QuickPlex SQ 120MM (MSD). During all incubation steps, plates were sealed with adhesive film and shaken at 700 rpm and 25 °C.

### Antigen-dependent quantification of anti-Spike IgG subclass concentrations

For antigen-dependent quantification of the anti-Spike IgG subclass concentrations, we generated calibration curves with different monoclonal antibodies of the four IgG subclasses that all target the same epitope of SARS-CoV-2 Wuhan Hu-1 Spike [6]. Herein, the assay was performed as described above, however, instead of using sera, wells were incubated with different concentrations of the monoclonal anti-Spike antibodies.

### Antigen-independent quantification of anti-Spike IgG subclass concentrations

For antigen-independent quantification of the concentrations of previously characterized anti-Spike IgG subclasses, obtained electrochemiluminescence signals were normalized to calibration curves that we generated for each secondary antibody. Herein, we reconstituted the Fc proteins of all four IgG subclasses (Acro Biosystems, Cat.: IgG1 Fc FCC-H5214, IgG2 Fc IG2-H5206, IgG3 Fc IG3-H5200, IgG4 Fc IG4-H5205) in deionized water and diluted them in phosphate buffered saline (PBS) with 0.03% Triton X-100. Subsequently, a 96-well plate with small spots (MSD, Cat.: L45XA) was coated with 1 µL/well of each Fc fragment at 11 different concentrations. The unsealed plate was incubated overnight without shaking. Following antigen coating, the plate was blocked with 150 µL/well blocking solution for 30 min and subsequently washed three times with 150 µL/well wash buffer. Biotinylated subclass-specific antibodies were added to the respective wells at the same concentrations that we used for serum IgG subclass characterization (see above). The plate was incubated for 1 h. After washing three times with 150 µL/well washing buffer, 25 µL/well SULFO-tag streptavidin was added at 0.2 µg/mL in diluent, and the plate was incubated for 1 h. The plate was washed again three times before 150 µL/well of read buffer was added to each well. Measurements were conducted on a MESO QuickPlex SQ 120MM (MSD). If not stated otherwise, all incubation steps were performed at 25 °C with sealed plates and shaking at 700 rpm. Total anti-Spike RBD IgG antibody concentrations were calculated as the sum of all IgG subclass-specific concentrations.

### Study cohort

For the validation of our assay, we utilized sera from ten healthy individuals (*n* = 10) who were part of a well-characterized study cohort [6]. All participants were immunized three times with the mRNA vaccine BNT162b2. We considered them SARS-CoV-2 infection-naïve as they reported no COVID-19 history and tested negative for anti-SARS-CoV-2 nucleocapsid antibodies at both sampling timepoints [6]. Individuals were sampled by median 15 days (IQR 14–18) after the second vaccination and 29 (IQR 19–41) days after the third vaccination, respectively. The cohort characteristics are summarized in Supplementary Table 1.

### Data analysis

Data and statistical analyses were performed in R statistical software (v4.2.2) and GraphPad Prism (v10.1.2). In R, the following packages were used: *psych* (2.4.12), *FSA* (0.9.6) and *tidyverse* (2.0.0). Continuous data was analyzed for statistically significant differences between groups of interest by the (paired) Wilcoxon rank sum test (2 groups) or the Friedman test (> 2 groups). In case a Friedman test was significant, we performed Nemenyi’s post hoc tests for all possible comparisons. For correlation analysis Spearman rank coefficients were calculated. Calibration curves for antigen-dependent and -independent quantification were generated in Prism (v10.1.2, GraphPad) using a 4-parameter logistic fit and 1/y² weighting. These curve fits were solved for the variable y (concentration) and used to calculate the anti-Spike RBD IgG subclass-specific antibodies of each sample. The final concentration was calculated by multiplying the results with the respective dilution factors. Figures were edited in Affinity Publisher (v2.4.0, Serif) to enhance their clarity and visual appearance.

## Results

### Assay establishment and generation of calibration curves

To quantify SARS-CoV-2 variant-specific immunoglobulin G (IgG) subclasses 1 to 4 in human sera we developed a multiplex assay using electrochemiluminescent (ECL) technology. We utilized two types of commercially available 96-well plates. The wells of the first type contained six different spots, each of which was pre-coated with full-length Spike antigen from one of the following SARS-CoV-2 variants: Wuhan Hu-1, Alpha, Beta, Gamma, Delta and Omicron BA.1. The wells of the second plate type contained nine spots that were pre-coated with the receptor binding domain (RBD) of the following SARS-CoV-2 variants: Wuhan Hu-1, Alpha, Beta, Delta, as well as Omicron sub-lineages BA.1, BA.2, BA.2.12.1, BA.2.75, and BA.4/BA.5.

**Fig. 1:**
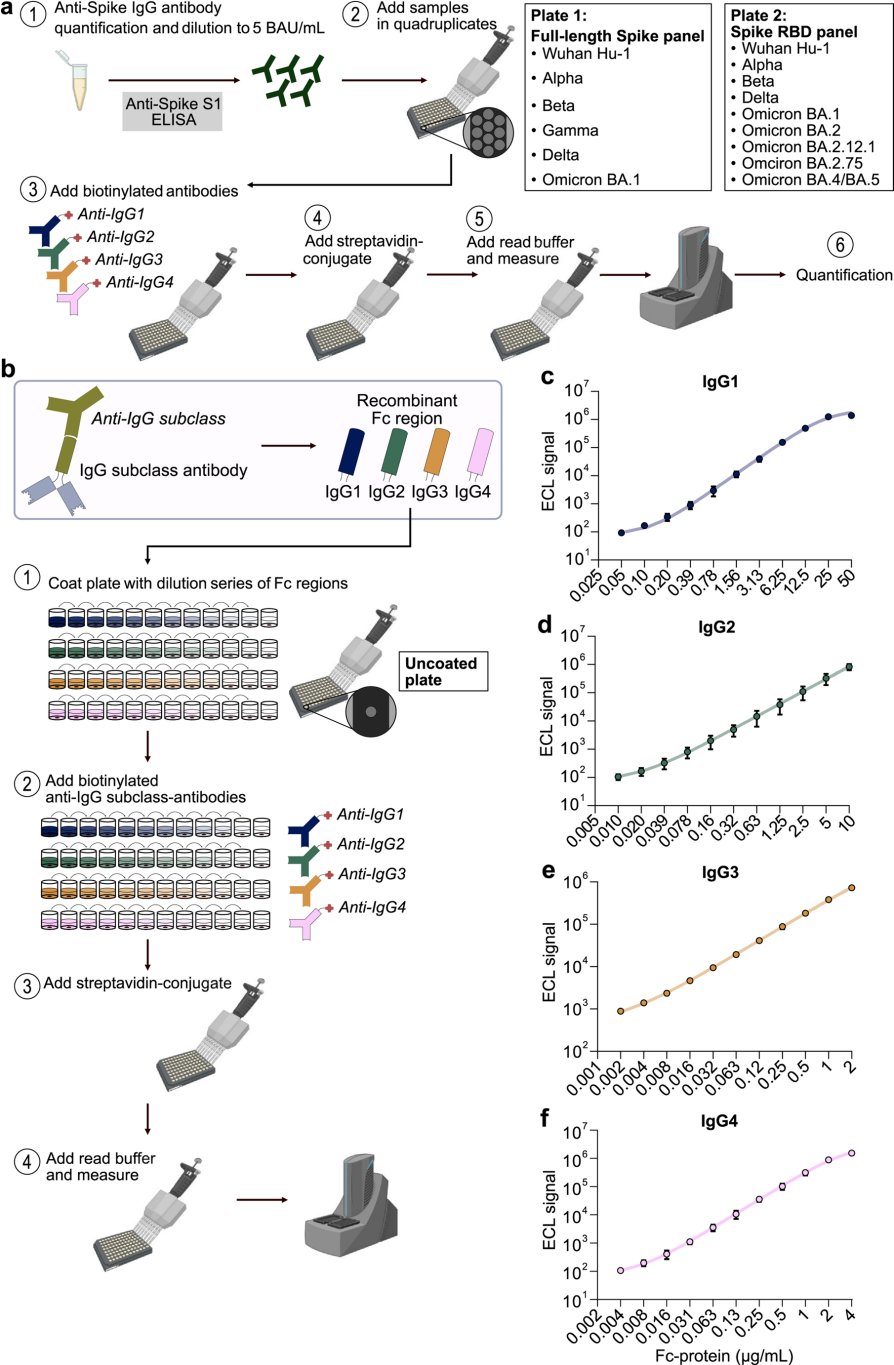
Assay workflow and generation of calibration curves for antigen-independent quantification. **a** Assay workflow. Total anti-Spike IgG antibody concentrations in sera were quantified and samples were diluted to 5 BAU/mL. Samples were incubated in quadruplicates on plates, for which each well was coated with Spike from different SARS-CoV-2 variants. Secondary antibodies against one of the four IgG subclasses were added to each well of the quadruplicates. After addition of streptavidin-conjugate, read buffer was added and signals were measured. **b** Antigen-independent quantification. The secondary anti-IgG subclass specific antibodies bind the Fc regions of the respective primary antibodies. Therefore, plates were coated with different concentrations of the Fc fragments of each IgG subclass to generate calibration curves for secondary antibody binding. After incubation with biotinylated IgG-subclass-specific secondary antibodies, streptavidin-conjugate and read buffer were added and signals were measured. **c–f** Calibration curves for antigen-independent quantification. The electrochemiluminescence (ECL) signals are depicted by the concentrations of the Fc regions of IgG1 **(c)**, IgG2 **(d)**, IgG3 **(e)** and IgG4 **(f)**. Dots and error bars represent the mean and standard deviation from three independent experiments, each of which contained two technical replicates. Error bars are shown for each data set, but in many cases error bars are not visible as the error is too small. Calibration curves were generated using a four-parameter fit. Parts of panels **a** and **b** were created in BioRender

The protocol to measure SARS-CoV-2 variant-specific anti-Spike IgG subclass signals consisted of six steps (Fig. 1a): (I) We measured the anti-Spike S1 IgG antibody concentration in each serum sample using a commercially available assay and subsequently diluted each sample to a defined anti-Spike antibody concentration. (II) The diluted sera were incubated in quadruplicates on the antigen-coated plate (one well per anti-IgG subclass detection antibody). (III) We added IgG subclass-specific, biotinylated secondary detection antibodies. (IV) The wells were then incubated with reagent-labeled streptavidin. (V) Subsequently, we added reagent buffer, and (VI) quantified the electrochemiluminescence signal on the appropriate reader. Using the first type of plates coated with full-length Spike proteins, unfortunately, lead to excessive binding of streptavidin making quantification impossible (suppl. Figure 2a). However, using the second type of plates coated with the RBD domains of the different Spike variants, we successfully obtained signals with low background. We, therefore, used the plates coated with different Spike RBD domains for all subsequent measurements.

The detected signal strength in this assay is not only dependent on the quantity and quality of anti-Spike RBD binding antibodies in the serum specimens but also on the binding properties of the secondary detection antibodies. To enable inter-IgG subclass and inter-SARS-CoV-2 variant comparisons from signals obtained with different detection antibodies we generated calibration curves. Two alternative approaches were tested: First, we explored normalization to signals of defined concentrations of recombinant, monoclonal antibodies of each IgG subclass that all target the same epitope of Wuhan Hu-1 Spike [6]. However, these antibodies showed reduced binding to the Spike RBD of Alpha and no binding to that of Beta, Delta and any Omicron variant making quantification of non-Wuhan Hu-1-specific antibody responses non-feasible with this technique (suppl. Figure 2b–e).

Therefore, we developed a second approach, in which we generated calibration curves by quantifying the signal of the detection antibodies after binding to defined concentrations of subclass-specific Fc fragments. Herein, we assumed that the binding of the secondary detection antibodies to the subclass-specific Fc fragments would be comparable to their binding to the antigen-bound primary antibodies from sera. In this calibration assay, the normalization of the signal is uncoupled from the viral antigen, against which the serological immune response is directed, i.e., SARS-CoV-2 Spike RBD. For this approach, which we termed “antigen-independent”, we coated previously uncoated plates with different amounts of purified, recombinant Fc fragments of each IgG subclass. The subclass-specific secondary detection antibodies were added to these plates at the same concentrations as in the assay that was used to quantify subclass-specific anti-Spike IgG responses in serum specimens. The calibration curve assays were completed using the same steps (IV – VI) and reagent concentrations as in the assay used for the serum samples (Fig. 1b). Performing this calibration assay, we were able to generate reliable calibration curves for the quantification of subclass- and variant-specific anti-Spike antibody signals (Fig. 1c–f). These calibration curves show the dependency of the detection antibody signals to the amount of coated subclass-specific Fc fragments and were used for the quantification of the anti-Spike RBD IgG subclass signals in all serum samples measured through interpolation.

### Assay validation and performance evaluation

To validate our assay and test its performance we utilized 20 serum specimens obtained from 10 COVID-19-naïve, healthy, vaccinated individuals whose SARS-CoV-2 Spike-specific IgG subclass composition had been previously characterized [6]. Individuals were sampled post second and post third vaccination with a COVID-19 mRNA vaccine.

**Fig. 2:**
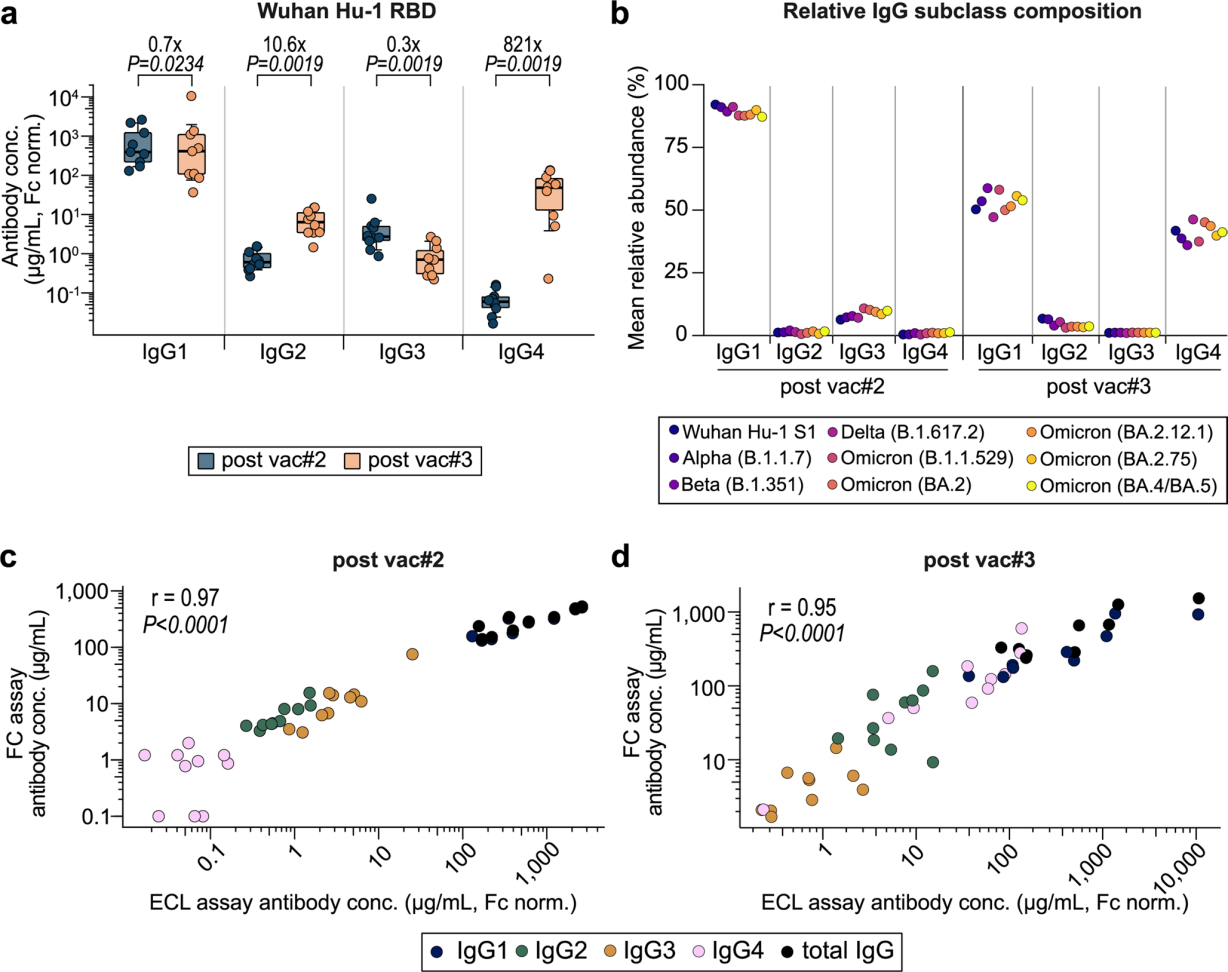
Evaluation of the assay’s performance in a cohort of infection-naïve vaccinees. **a** Wuhan Hu-1-specific anti-Spike RBD IgG subclass concentrations measured in ten infection-naïve individuals after the second (blue) and the third vaccination (orange) with an mRNA vaccine. Individual data points are depicted and box plots with medians, bounds between upper and lower quartiles as well as whiskers between the 10th and 90th percentiles. Differences between timepoints were analyzed for statistical significance using the paired Wilcoxon rank sum test. *P*-values and the median-fold differences comparing timepoints are reported. **b** Relative abundance of Spike RBD targeting, variant-specific IgG subclasses. Shown are the means from ten individuals measured after the second (left) and third vaccination (right). **c–d** Correlation analyses comparing results from the herein described method (ECL) with published data acquired from the same samples using a flow cytometry method (FC) after the second **(c)** and the third **(d)** vaccination (6). Spearman’s r and corresponding *p*-values are reported. Fc norm. – concentration obtained by normalization to Fc calibration curves

Comparing Wuhan Hu-1 Spike RBD-specific-IgG subclasses after the third vaccination to those after the second, we found a reduction of the median IgG1 and IgG3 levels (IgG1: 0.7-fold, *p* = 0.0234; IgG3: 0.3-fold, *p* = 0.0019, Fig. 2a). IgG2 and IgG4 levels, in contrast, showed a 10.6-fold (*p* = 0.0019) and 821-fold (*p* = 0.0019) increase, respectively, in the same comparison. Similar kinetics of slightly decreasing IgG1 and IgG3 concentrations and increasing levels of IgG2 and, especially, IgG4 were found for the RBD-specific responses against other SARS-CoV-2 variants (suppl. Figure 3).

Next, we analyzed the mean relative rates of anti-Spike RBD-IgG antibodies of the four different subclasses across all nine tested SARS-CoV-2 variants (Fig. 2b). After the second COVID-19 vaccination, the relative abundance of all subclasses was similar between the variants. IgG1 constituted on average 89.3% of all anti-Spike RBD antibodies, ranging from 87.2% in Omicron BA.4/BA.5 to 92.1% in Wuhan Hu-1. IgG3 antibodies accounted on average for 8.5% with a minimum of 6.3% in Wuhan Hu-1 and a maximum of 10.8% in Omicron B.1.1.529. IgG2 and IgG4 antibodies were the least prevalent in all variants, accounting for 1.3% (0.6% in Omicron B.1.1.529–2.0% in Beta) and 0.9% (0.4% in Wuhan Hu-1–1.3% in Omicron BA.4/BA.5), respectively. After the third vaccination, relative abundance of IgG subclasses was still comparable across variants. However, IgG1 and IgG4 responses showed slightly increased variability. On average, 53.6% (47.3% in Delta − 58.8% in Beta) of all anti-Spike RBD antibodies were IgG1. IgG4 accounted on average for 41.2% of all anti-Spike RBD antibodies and was most prevalent in Delta with 46.4% and least prevalent in Beta with 36.1%. The relative IgG2 abundance ranged from 3.1% in Omicron B.1.1.529 to 6.7% in Wuhan Hu-1. IgG3 accounted on average for 1.2% of all specific antibodies with a minimum of 1.0% in Delta and a maximum of 1.2% in Omicron B.1.1.529.

Comparing variant-specific IgG subclass concentrations, we found that both after the second and after the third vaccination, responses were strongest against Wuhan Hu-1 across all subclasses (suppl. Figure 4). IgG subclass concentrations of antibodies targeting Alpha and Delta were comparable to those targeting Wuhan Hu-1, and those against Beta were reduced by trend. IgG subclass concentrations of antibodies specific to the Omicron variants were significantly reduced in all instances compared to antibody responses to Wuhan Hu-1. The anti-Spike RBD IgG subclass levels among the different Omicron variants were, however, comparable.

Finally, we performed correlation analyses of the IgG subclass composition of Wuhan Hu-1 Spike-specific antibodies comparing the results obtained with our newly developed anti-Spike RBD subclass quantification assay to those measured previously in the same samples via a flow cytometry-based method [6]. At both timepoints, results from our assay correlated strongly and significantly with the published results (post 2nd vacc.: *r* = 0.97, *p* < 0.001; post 3rd vacc.: *r* = 0.95, *p* < 0.001) (Fig. 2c, d).

## Discussion

Here, we describe a novel multiplex assay for the quantification of IgG subclass compositions of anti-Spike RBD antibodies against multiple SARS-CoV-2 variants. Our method relies on an antigen-independent quantification technique, requires low sample volumes of less than 10 µL per donor and, due to multiplexing, the IgG subclass-specific antibody responses against up to ten different antigens can be measured in one well. The assay allows for up to 200 serum specimens being evaluated for their SARS-CoV-2 variant-specific subclass compositions within approximately 4.5 h when being performed by a single person. As it exclusively utilizes commercially available reagents and is carried out in 96-well plates, the method can be automated to a high degree.

We validated our technique with sera from a well-described, longitudinally sampled cohort that was previously analyzed for their anti-Spike IgG subclass compositions by a flow-cytometric method [6]. In case of anti-Wuhan Hu-1 Spike IgG subclass quantifications, the assays showed a strong and statistically highly significant correlation. Corroborating previous findings, we observed decreasing levels of antigen-specific IgG1 and IgG3 after the third mRNA vaccination while those of IgG2 and, especially, IgG4 increased [6–12]. Additionally, we found that anti-Spike RBD IgG subclass kinetics were comparable between the nine different SARS-CoV-2 variants tested. In line with our results, it was recently reported that the kinetics of total IgG antibodies against the Spike RBD of Delta, Omicron BA.2 and BA.4/BA.5 variants were comparable to those against Wuhan Hu-1 Spike RBD in individuals who received at least three immunizations with an mRNA vaccine [15].

To our knowledge, the herein described assay is the first to quantify anti-Spike RBD IgG subclass concentrations against several SARS-CoV-2 variants in multiplex that solely relies on commercially available reagents. While the relative composition of Spike RBD-specific IgG subclasses was similar comparing different virus variants, we observed a decrease among all IgG subclass levels against more recent virus variants. All participants of the study cohort were immunized with a vaccine encoding ancestral Spike and were infection-naïve. Conceivably, the observed reduction in binding to “more evolved” Spike proteins is a testament of the frequently reported immune evasion of later virus variants [20, 21]. Not only antibody-dependent neutralization, but also other IgG-mediated effector functions against recent SARS-CoV-2 variants may, thus, be impaired. This includes IgG4-promoted Fcγ receptor-dependent phagocytosis which was recently shown to be of particular importance in the immune responses to SARS-CoV-2 Omicron [14]. To broaden our knowledge on humoral protection against COVID-19, future studies should focus on deciphering SARS-CoV-2 variant-specific IgG subclass compositions, concentrations and effector functions in larger cohorts that include individuals vaccinated more than three times, in part, with Omicron-adapted vaccines as well as persons who experienced breakthrough infections. Assays like the one described in this manuscript may be valuable to conduct such research. Connecting the quantification of anti-Spike RBD IgG subclass responses to antibody-mediated effector functions measured in serum specimens by using assays such as ours may aid in the discovery of novel immunological phenomena. Furthermore, it could add evidence to previous observations, including the reduced complement activation, phagocytic activity and antibody dependent cellular toxicity associated with high levels of antigen-specific IgG4 [6, 8, 14], as well as the increased COVID-19 risk in individuals with higher anti-SARS-CoV-2 Spike IgG4 and IgG2 concentrations [15].

Due to the design of their calibration methods, assays, such as the one described here, are often unreliable to measure the real and accurate concentration of antigen-specific antibodies in serum specimens. Indeed, the anti-Spike RBD subclass concentrations that we obtained in our anti-Spike RBD IgG subclass quantification assay by normalization to the Fc fragment concentrations are approximately 10- to 100-fold higher than reported by others and, thus, physiologically implausible [6, 9, 12]. The purified Fc fragments used in the calibration assay are substantially smaller than full-length antibodies. These Fc-fragments will, therefore, differ in their molarity from full-length IgGs when diluted in similar milligram per milliliter concentrations. Additionally, the Fc-fragments potentially adhered more efficiently to the assay plate than full-length antibodies, both in terms of quantity and exposure of their antigenic regions, leading to a downstream signal increase. Taken together, this may cause an overestimation of the antigen-specific serum IgG subclass concentrations measured with our technique. For the assessment of the relative antigen-specific IgG subclass compositions and inter-variant comparisons, which are the main purposes of our method, this overestimation is, however, inconsequential. Our study cohort was small and consisted solely of infection-naïve vaccinees. In addition, we did not include quantification of the IgG-specific anti-SARS-CoV-2 RBD antibody responses against the most recent SARS-CoV-2 variants in this study. Thanks to our antigen-independent Fc normalization approach however, the assay can be easily adapted for measuring responses to Spike RBD from such recent virus variants. Another limitation of our method is that it only quantifies IgG subclass responses against the RBD of Spike instead of the full-length protein. RBD is, however, the most immunogenic region of Spike [22, 23] and, moreover, the most important domain for eliciting neutralizing activity and other antibody-mediated effector functions [22, 24]. Hence, we are confident that our newly developed assay covers the most essential IgG subclass response to assess humoral immunity to COVID-19.

Throughout the pandemic, serological assays were relevant for identifying potential COVID-19 risk factors [25–28], to evaluate the success of vaccination [21, 29, 30] and for surveilling disease outbreaks in healthcare cohorts [28, 31]. Differentiation between the four different subclasses of anti-Spike IgG antibodies and investigation of their role in anti-viral immunity has grown in importance after reports that repeated immunization with mRNA vaccines causes an unprecedented increase in Spike-targeted, non-inflammatory IgG4 responses [6–13]. Due to being easy-to-establish and having the potential to be set up for high-throughput measurements, our assay is suitable for investigating such SARS-CoV-2 anti-Spike IgG subclasses in larger cohorts. This differentiates it from existing ELISA- or flow cytometry-based assays. Our approach enables quantification of variant-specific antibody responses in multiplex, which may help contribute to our understanding of the immunological interplay between immunizations with original and adapted mRNA vaccines and breakthrough infections with recent SARS-CoV-2 variants. The fact that our assay relies on antigen-independent quantification makes it easy to adapt to emerging virus variants and, potentially, capable of quantifying IgG subclasses specific to other pathogens.

## Supplementary Information

Below is the link to the electronic supplementary material.

Supplementary Material 1

Supplementary_Material 2

## Data Availability

All data supporting the findings of this study are available within the paper and its Supplementary Information.
