# Supplementary material for: Simultaneous quantification of anti-Spike IgG subclasses against various SARS-CoV-2 variants using a multiplex serological assay
Source: Med Microbiol Immunol. 2026 May 22;215(1):16. doi: 10.1007/s00430-026-00878-y (PMC13197274; doi:10.1007/s00430-026-00878-y)
Supplement: Supplementary file 2 — Supplementary_Material 2 [file 430_2026_878_MOESM2_ESM.pdf]

## Supplementary material

# Simultaneous quantification of anti-Spike IgG subclasses against various SARS-CoV-2 variants using a multiplex serological assay

Marie L. Bischof, Pascal Irrgang, Matthias Tenbusch, Oliver T. Keppler, Paul R. Wratil

# Supplementary Tables

**Supplementary Table 1**: Characteristics of the study cohort.

| | |
|---|---|
| Vaccinations | BNT162b2 mRNA, 3x |
| Number of Participants | 10 |
| Age in years, median (IQR) [range] | 37 (25-53) [25-59] |
| Sex n (%) female | 5 (50) |
| Sex n (%) male | 5 (50) |
| Time interval between vaccinations in days, median (IQR) [range] | |
| first to second | 23 (21-24) [21-25] |
| second to third* | 211 (197-232) [170-251] |
| time interval from vaccination to blood sampling in days, median (IQR) [range] | |
| post second | 15 (14-18) [14-20] |
| post third * | 29 (19-41) [15-61] |

* for one sample the exact date of the 3rd vaccination is missing

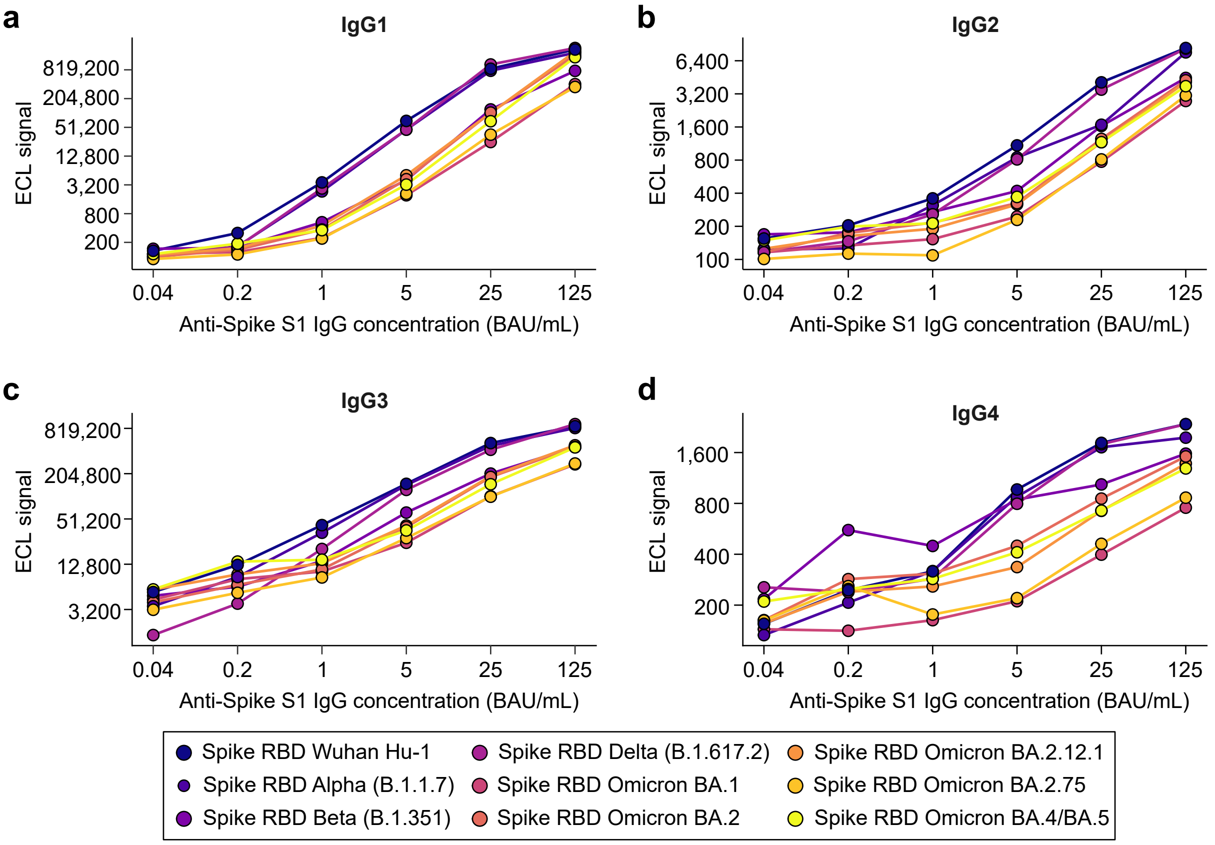


**Supplementary Figure 1:** The signal strengths measured in the anti-Spike RBD IgG subclass characterization assay depend on the input concentration of anti-Spike. The sera collected from all 10 participants after the second mRNA vaccination were pooled and diluted in sample buffer to different anti-Spike concentrations before being measured in the anti-Spike RBD IgG subclass characterization assay. Depicted are the signal strengths measured in the assay by the anti-Spike S1 concentration of the pooled sera in binding antibody units per mL (BAU/mL) for IgG1 **(a)**, IgG2 **(b)**, IgG3 **(c)** and IgG4 **(d)**.

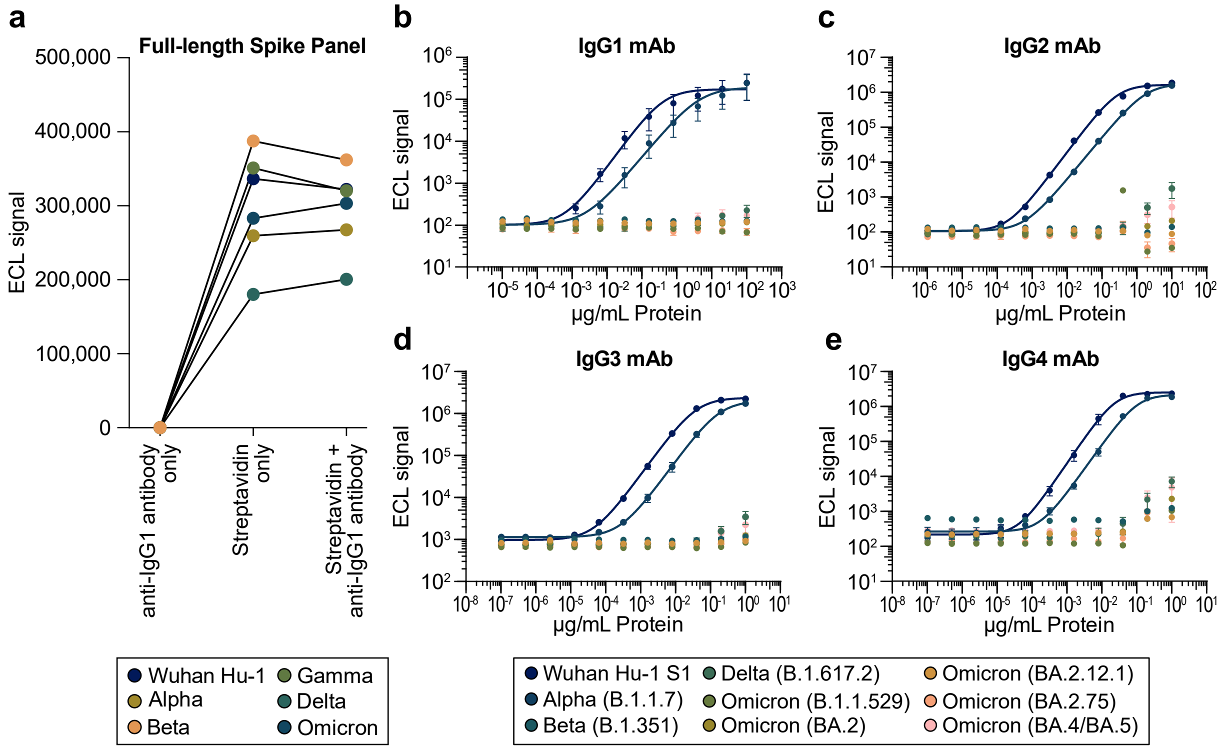


**Supplementary Figure 2: Evaluation of the quantification of anti-full-length Spike IgG subclasses and antigen-dependent quantification**

**(a)** Electrochemiluminscence (ECL) signals acquired using plates coated with full-length Spike from different SARS-CoV-2 variants treated with either only anti-IgG1 antibody, only streptavidin or anti-IgG1 plus streptavidin. **(b-e)** Calibration curves for antigen-dependent quantification using recombinant, monoclonal antibodies of IgG subclass IgG1 **(b)**, IgG2 **(c)**, IgG3 **(d)** and IgG4 **(e)** that all target the same Spike epitope. The ECL signals are depicted by the antibody concentrations. Dots and error bars represent the mean and standard deviation from three independent experiments. In case of Wuhan Hu-1 and Alpha, calibration curves were generated using a four-parameter fit.

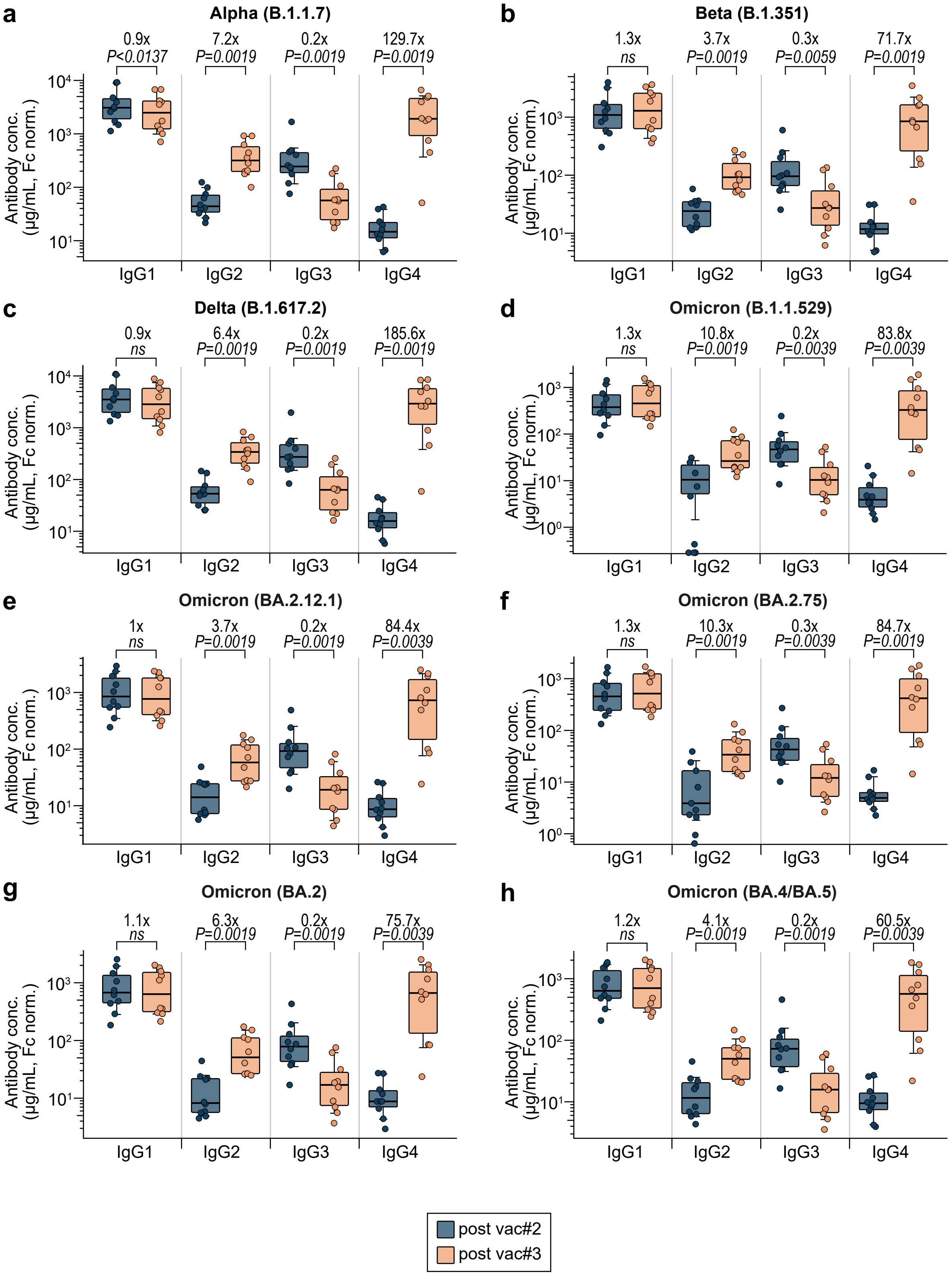

a
Alpha (B.1.1.7)
0.9x P<0.0137
7.2x P=0.0019
0.2x P=0.0019
129.7x P=0.0019
b
Beta (B.1.351)
1.3x ns
3.7x P=0.0019
0.3x P=0.0059
71.7x P=0.0019
c
Delta (B.1.617.2)
0.9x ns
6.4x P=0.0019
0.2x P=0.0019
185.6x P=0.0019
d
Omicron (B.1.1.529)
1.3x ns
10.8x P=0.0019
0.2x P=0.0039
83.8x P=0.0039
e
Omicron (BA.2.12.1)
1x ns
3.7x P=0.0019
0.2x P=0.0019
84.4x P=0.0039
f
Omicron (BA.2.75)
1.3x ns
10.3x P=0.0019
0.3x P=0.0039
84.7x P=0.0019
g
Omicron (BA.2)
1.1x ns
6.3x P=0.0019
0.2x P=0.0019
75.7x P=0.0039
h
Omicron (BA.4/BA.5)
1.2x ns
4.1x P=0.0019
0.2x P=0.0019
60.5x P=0.0039
Antibody conc. (µg/mL, Fc norm.)
IgG1
IgG2
IgG3
IgG4
post vac#2
post vac#3

**Supplementary Figure 3: Comparison of variant-specific anti-Spike RBD IgG subclass concentrations between sampling timepoints**

Concentrations of the four IgG subclasses against the Spike RBD of SARS-CoV-2 variants Alpha **(a)**, Beta **(b)**, Delta **(c)** as well as Omicron B.1.1.529 **(d)**, BA.2.12.1 **(e)**, BA.2.75 **(f)**, BA.2 **(g)**, and BA.4/BA.5 **(h)** are shown acquired from ten infection-naïve individuals after the second and the third vaccination with an mRNA vaccine. Individual data points are depicted and box plots with medians, bounds between upper and lower quartiles as well as whiskers between the 10th and 90th percentiles. Differences between timepoints were analyzed for statistical significance using the paired Wilcoxon rank sum test. P-values and the median-fold differences comparing timepoints are reported. Fc norm. – concentration obtained by normalization to Fc calibration curves

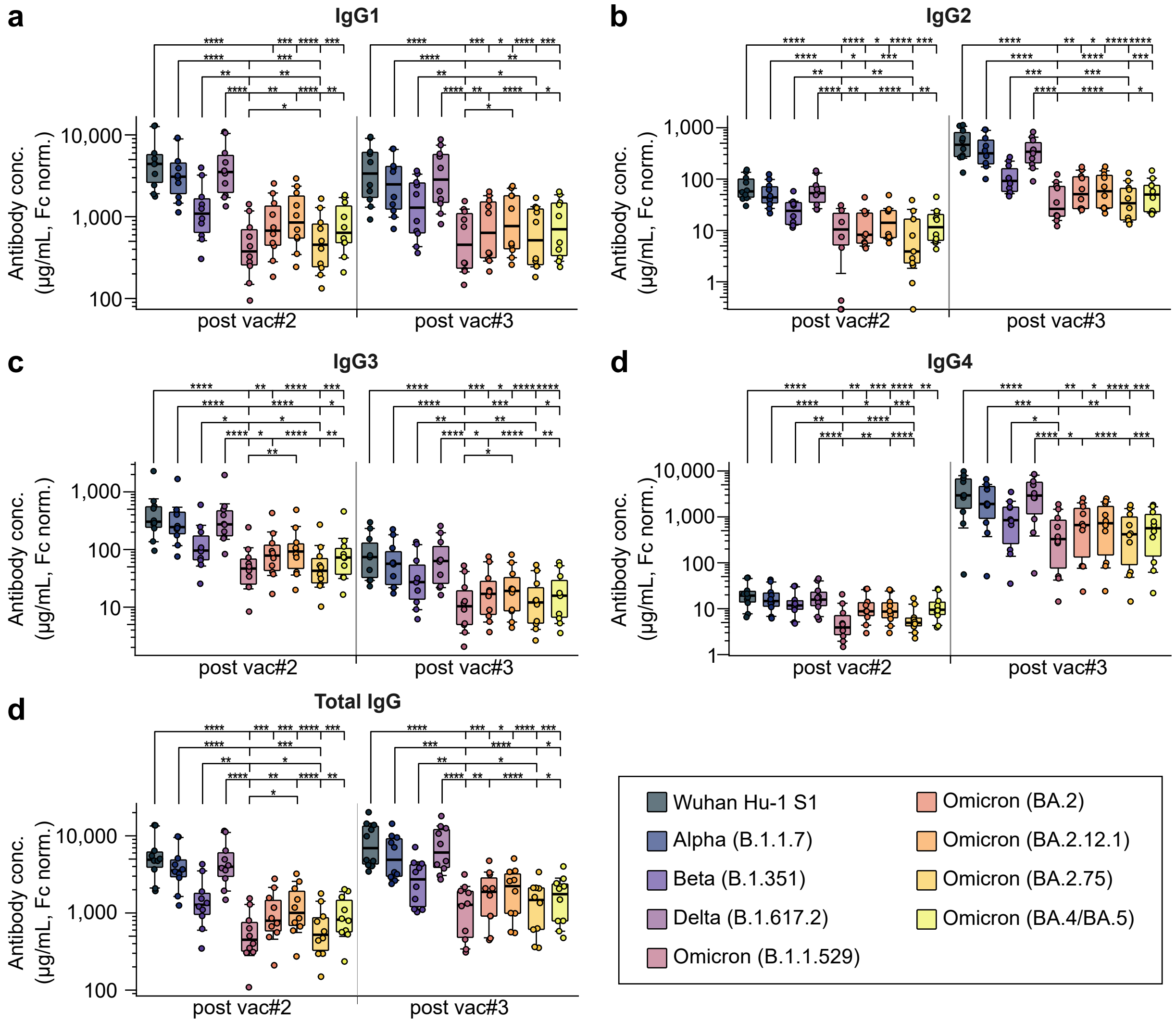


**Supplementary Figure 4: Comparison of IgG subclass concentrations between antibodies specific to the Spike RBD of different SARS-CoV-2 variants**

Concentrations of IgG1 **(a)**, IgG2 **(b)**, IgG3 **(c)**, IgG4 **(d)** and total IgG **(e)** antibodies specific for Spike RBD of SARS-CoV-2 variants Wuhan Hu-1, Alpha, Beta, Delta as well as Omicron B.1.1.529, BA.2.12.1, BA.2.75, BA.2, and BA.4/BA.5 in ten individuals after the second and the third vaccination with an mRNA vaccine. Individual data points are depicted and box plots with medians, bounds between upper and lower quartiles as well as whiskers between the 10th and 90th percentiles. Differences between variant-specific antibody concentrations were analyzed for statistical significance using the Friedman test and the post hoc Nemenyi test. Brackets indicate the pairwise comparisons. P-values are reported as asterisks (*P≤0.05*: *, *P≤0.01*: **, *P≤0.001*: ***, *P≤0.0001*: ****) in case statistically significant differences were detected between groups. Fc norm. – concentration obtained by normalization to Fc calibration curves.
